# Imaging-based machine learning for the diagnosis and prognosis of uveal melanoma: a systematic review and meta analysis

**DOI:** 10.1007/s00417-026-07230-3

**Published:** 2026-04-18

**Authors:** Andres Bravo-Gonzalez, Pablo Dominguez-Ruiz, Maria J. Buitrago-Gonzalez, Bernardo Bach, Zhi Chen, Daniel Suarez, Mateus Pimenta Arruda, Rian Vilar Lima, Giulia Steuernagel Del Valle, Mariana Tosato Zinher, Carlos Eduardo de Menezes e Souza Filho, Pedro F. Salazar

**Affiliations:** 1 Universidad del Rosario, Escuela de Medicina y Ciencias de la Salud, Bogotá, Colombia; 2 Epidemiology MSc Programme, London School of Hygiene and Tropical Medicine, London, UK; 3 Clínica CardioVID, Medellín, Colombia; 4 Universidad CES, Medellín, Colombia; 5 Ophthalmology Division, Hospital de Clínicas (Universidade Federal do Paraná), Curitiba, Brazil; 6 Iowa Institute for Biomedical Imaging, University of Iowa, Iowa City, IA 52242 USA; 7 Fundación Oftalmológica Nacional, Bogotá, Colombia; 8 Department of Ophthalmology and Visual Sciences, Universidade Federal de São Paulo, São Paulo, Brazil; 9 Health Science Center, Universidade de Fortaleza, Fortaleza, Brazil; 10 Hospital de Olhos do Paraná, Curitiba, Brazil; 11 Ophthalmology Division, Santa Casa de Misericórdia de Belo Horizonte, Belo Horizonte, Brazil

**Keywords:** Uveal melanoma, Intraocular tumors, Artificial Intelligence, Machine learning, Ocular oncology

## Abstract

**Background:**

Uveal melanoma (UM) is the most common primary intraocular malignancy in adults and carries a high risk of metastasis and poor prognosis when diagnosed late. Distinguishing UM from benign choroidal nevi remains challenging due to overlapping imaging features. Machine learning (ML) and deep learning (DL) models have emerged as tools to improve diagnostic accuracy and prognostic prediction, but their generalizability and clinical readiness remain uncertain.

**Purpose:**

To systematically review and meta-analyze the performance of published ML/DL algorithms using ocular imaging on detecting and predicting prognosis of UM.

**Methods:**

A systematic search was conducted in PubMed, Scopus, Web of Science, Embase, and IEEE Xplore up to June 2025 for English and Spanish publications since 2012. Eligible studies applied ML/DL to ocular imaging and reported diagnostic or prognostic accuracy metrics. Risk of bias was assessed with QUADAS-2, and pooled sensitivity and specificity were estimated using a random-effects meta-analysis, with subgroup analyses by imaging modality (fundus-only vs. ultrasound-based imaging, with or without an additional modality). For diagnostic studies, we additionally synthesized positive and negative likelihood ratios along with diagnostic odds ratios (DOR) and conducted hierarchical summary receiver operating characteristic curve (ROC) (Reitsma) analyses stratified by modality.

**Results:**

Thirteen diagnostic studies met inclusion criteria. Most were retrospective, single-center cohorts using fundus photography, while others employed ultrasound (US), ultra-widefield (UWF), OCT, or autofluorescence. Ten studies used DL architectures, mainly convolutional neural networks or transformers. Pooled sensitivity was 78.21% and specificity 94.28%. Fundus-based models showed lower specificity (87.70%) than US-based models (98.16%). AUC values were consistently high; HSROC AUC was 0.912 (fundus) vs. 0.984 (US; Δ0.073, bootstrap 95% CI − 0.101 to 0.143). The largest modality separation was for LR+ (39.48 vs. 6.16; *p* < 0.0001), with higher DOR for US (225.25 vs. 27.89; *p* = 0.0102), while LR − was similar. Six prognostic studies (up to 4,600 patients) reported AUCs between 0.71 and 0.92 for predicting metastasis, survival, or enucleation, though all lacked external validation.

**Conclusions:**

ML and DL models show strong diagnostic performance and emerging prognostic value in UM. However, reproducibility and real-world validation remain limited. New foundation models such as RETFound and VisionFM, trained on large multimodal eye datasets, could improve standardization, explainability, and cross-center generalization. As these models evolve, they have the potential to become essential tools in clinical practice, accelerating the translation of AI into reliable, routine implementation in ocular oncology. (PROSPERO ID: CRD42025643874).

**Supplementary Information:**

The online version contains supplementary material available at 10.1007/s00417-026-07230-3.

## Key messages

*****What is known***:**

- Distinguishing uveal melanoma from choroidal nevus is challenging; ML/DL models have shown promise but concerns persist regarding generalizability and real-world readiness.

*****What is new***:**

- In a meta-analysis of 13 diagnostic studies, pooled performance was sensitivity 78.21% and specificity 94.28%; ultrasound-based models achieved higher specificity (98.16%) than fundus photograph-based models (87.70%).
- Evidence from six prognostic studies (up to 4,600 patients) shows AUC 0.71–0.92 for metastasis/survival/enucleation, but external validation is lacking and methodological heterogeneity is substantial.
- Multicenter external validation and adoption of foundation models (e.g., RETFound) are essential to improve robustness, explainability, and cross-center generalization in future AI applications for ocular oncology.

**Supplementary Information:**

The online version contains supplementary material available at 10.1007/s00417-026-07230-3.

## Introduction

### Uveal melanoma

Uveal melanoma (UM) is the most common primary intraocular malignancy in adults, with an incidence of approximately 5–10 cases per million annually in the United States and Europe [1, 2]. Most tumors originate in the choroid (90%), followed by the ciliary body and iris [2, 3]. Ocular ultrasonography is widely used to assess tumor dimensions, internal reflectivity, and extrascleral extension, while ancillary imaging such as fundus photography and optical coherence tomography (OCT) provides critical structural information.

The “To Find Small Ocular Melanoma” (TFSOM) mnemonic summarizes key features linked with malignant potential, including tumor thickness greater than 2 mm, subretinal fluid, visual symptoms, orange pigment, and proximity of the lesion margin to the optic disc. Variants of the mnemonic incorporate further parameters such as ultrasound hollowness, absence of a halo and absence of drusen, and large retrospective series have shown that the likelihood of transformation increases stepwise with the number of features present, with a 5-year transformation rate rising from 1% (no risk factors) to 55% (five risk factors) [4]. Complementary to this, the MOLES scoring system grades five features, mushroom shape, orange pigment, large size, enlargement, and subretinal fluid, assigning a score that stratifies lesions from benign nevus to probable melanoma [5]. Designed to be applied even without access to ultrasonography, MOLES has been shown to achieve near-perfect sensitivity for detecting lesions that require specialist referral, enabling its safe use in community or teleophthalmology settings [6].

The greatest limitations of detection acronyms center on their reliance on few clinical features that may be variably recognized and interpreted, as well as their limited integration of advanced imaging and molecular diagnostics [5]. In contrast, AI-based models are not limited to these manually selected parameters; they can analyze thousands of imaging patterns simultaneously, integrating subtle textural, spatial, and multimodal information beyond human perception. This capacity allows ML and DL approaches to capture the full complexity of lesion morphology and behavior, offering the potential for more objective, reproducible, and data-driven classification of UM [7].

Despite improvements in imaging techniques and local tumor control, up to 50% of patients develop metastatic disease, with poor survival once metastasis occurs [2, 3]. Therefore, early and accurate diagnosis is essential for guiding timely intervention and optimizing patient outcomes.

### Machine learning and deep learning models

Artificial intelligence (AI) models used in medical imaging and clinical decision-making can be broadly categorized along a spectrum that spans from general-purpose algorithms to highly specialized architectures [8].

AI models can be divided into two principal families: traditional machine learning (ML) algorithms and deep learning (DL) architectures [9]. Traditional ML algorithms, such as logistic regression, random forests (RF), gradient boosting methods like XGBoost and LightGBM, and ensemble techniques such as Extra Trees, are typically applied to structured data shown as variable specific values [10]. They are often used in settings where clinical variables have already been extracted or quantified [10].

In contrast, DL models are particularly well suited for unstructured data such as medical images. Within this domain, convolutional neural networks (CNNs) constitute the foundational architecture for most computer vision tasks [8]. Early CNNs such as AlexNet and VGG16 introduced layered convolutional structures that automatically learn spatial hierarchies of features from raw image pixels [11]. More advanced variants, such as ResNet (residual networks) and DenseNet (densely connected networks), addressed optimization challenges associated with increasing model depth [12].

Diagnostic and prognostic ML models serve distinct purposes in UM: diagnostic models aim to classify lesions as benign or malignant, often based on imaging features alone, while prognostic models predict clinical outcomes such as metastatic risk or survival, typically incorporating genomic, histopathologic, or longitudinal clinical data. This systematic review and meta-analysis aims to evaluate and explore the heterogeneity within the performance of ML models designed for UM at diagnosis and prognosis, separately. A PICO framework to understand both is displayed on Supplementary Table 1.

## Methods

### Study design

This systematic review and meta-analysis followed the Cochrane Handbook for Systematic Reviews of Diagnostic and Prognostic Models and adhered to the Preferred Reporting Items for Systematic Reviews and Meta-Analyses (PRISMA) guidelines [13]. The protocol was prospectively registered in PROSPERO (CRD42025643874).

### Search strategy and selection criteria

A comprehensive literature search was conducted on June 30th, 2025, using five major databases: PubMed, Scopus, Web of Science, Embase, and IEEE Xplore. (supplementary Table 2). The search was restricted to articles published in English or Spanish.

**Fig. 1:**
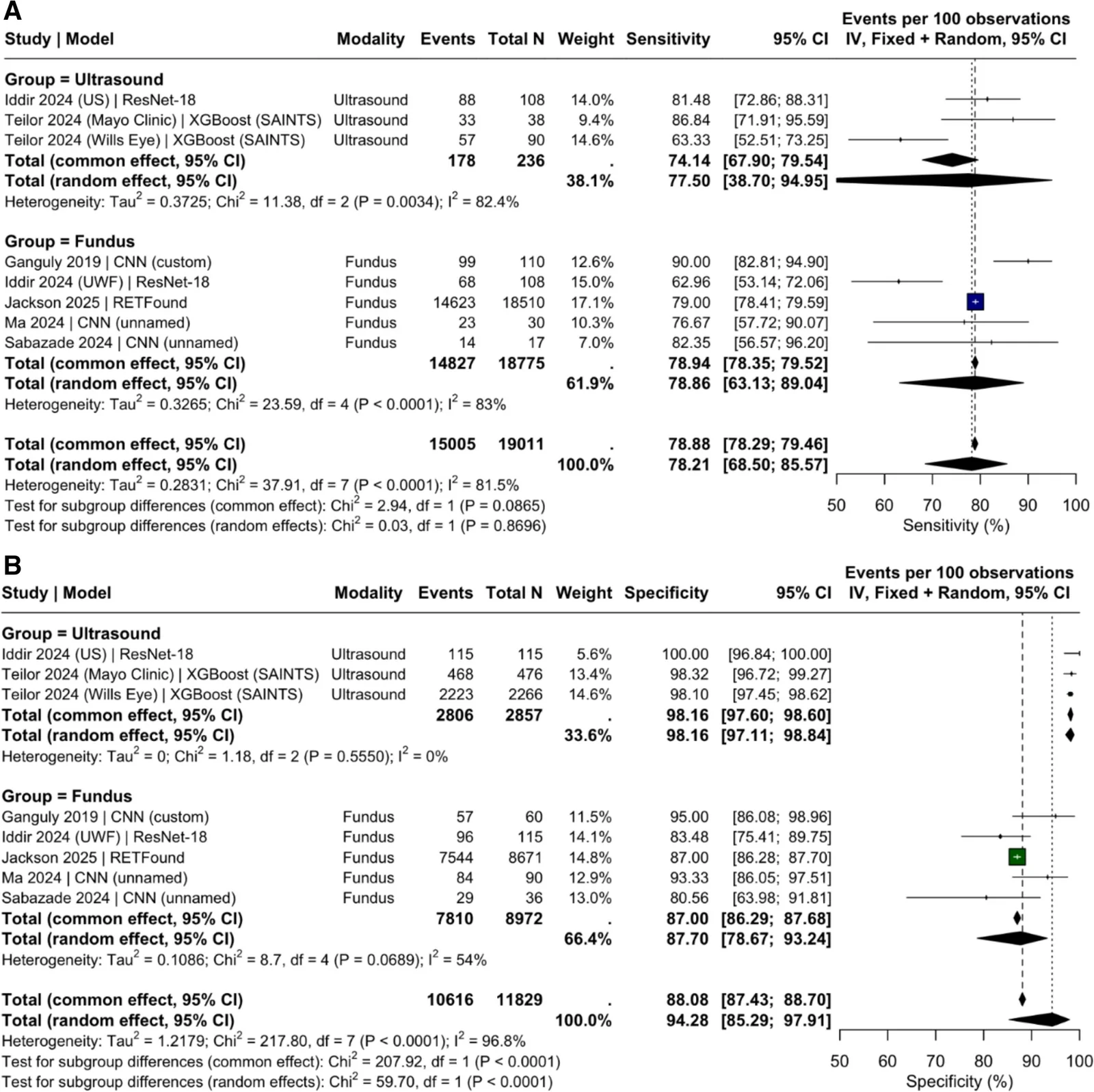
Subgroup analysis by imaging modality used as input in the training or validation model for (**A**) Sensitivity and (**B**) Specificity

Eligible studies included adult patients either undergoing evaluation for UM or with an established diagnosis (Fig. 1). We included studies applying ML/DL models to imaging data; prognostic studies using image-derived metrics (e.g., tumor thickness) were also included even when images were not directly analyzed. Included studies had to report diagnostic performance (e.g., sensitivity, specificity, AUC) and/or prognostic outcomes (e.g., metastasis-free survival) and use a clearly defined reference standard (expert imaging review, longitudinal follow-up, and/or histopathology). Studies based solely on genetic, transcriptomic, or molecular analyses without imaging inputs were excluded.

For study selection, three reviewers (AB-G, PD-R, and MJB-G) independently and blindly screened records, assessed full texts, extracted data and assessed the risk of bias; disagreements were resolved by consensus. We hand-searched reference lists of included studies but identified no additional eligible articles. No grey literature or other additional sources were searched.

### Data extraction and management

Extracted data included author, year, study objective, ML models, dataset size, performance metrics (sensitivity, specificity, AUC, accuracy, precision, recall, F1-score), and details of validation with internal or external datasets. If necessary, study authors were contacted to obtain missing or unclear data. When unable to collect all necessary data for specific subgroup analyses, incomplete datasets were omitted from these; if unable to collect any data to conduct any analyses, studies were excluded. Both number of images and number of patients were included when available but the unit of analysis to calculate diagnostic performance metrics was on an image-level regardless of the number of images each patient had to enhance comparability.

### Model selection

Models were selected based on a prioritized framework to ensure consistency and clinical relevance across studies. First, for each study, only one outcome was included per imaging modality. If multiple models were reported for the same modality and population, priority was given to the model with external validation and the most complete reporting of sensitivity and specificity.

Second, when studies reported results for distinct imaging modalities, such as US and UWF, one outcome per modality was retained. For example, in Iddir 2024, both US-based and UWF-based models were compared in the subgroup analysis.

Third, models were grouped into two predefined imaging categories: [1] Fundus photograph-based models and [2] US-based models.

### Meta-analysis and statistical methods

A diagnostic test accuracy meta-analysis was performed using hierarchical models for paired sensitivity and specificity, summarized with ROC curves. As a complementary approach, we also conducted univariate random-effects meta-analyses for sensitivity and specificity separately using the DerSimonian–Laird method with inverse-variance weighting to provide pooled estimates and 95% CI and to support forest plot visualization. We additionally pooled LR+, LR−, and DOR, and performed subgroup analyses by imaging modality. All analyses were conducted in R (version 4.5.1) using the ‘mada’, ‘meta’, and ‘metafor’ packages.

### Evaluation of heterogeneity and risk of bias

Heterogeneity across included studies was assessed both visually and statistically. We began by visually inspecting forest plots of sensitivity and specificity, as well as ROC space scatterplots, to evaluate the consistency of diagnostic performance estimates across studies. Statistical heterogeneity was quantified using the I² statistic and between-study variance (τ²) derived from the univariate and bivariate random-effects models. I² values above 50% were considered indicative of substantial heterogeneity.

To explore sources of heterogeneity, we planned a pre-specified subgroup analysis stratified by Imaging modality. Subgroup-specific estimates of pooled sensitivity and specificity were generated using separate bivariate random-effects models for each category. Due to the limited number of studies per subgroup in some cases, formal meta-regression was not conducted.

The risk of bias for diagnostic accuracy studies was assessed using the Quality Assessment of Diagnostic Accuracy Studies-2 (QUADAS-2) tool. This evaluates four domains: (1) patient selection, (2) index test, (3) reference standard, and (4) flow and timing. Each domain was judged as having low, high, or unclear risk of bias, with applicability concerns assessed in the first three domains [14].

For studies reporting prognostic models or risk stratification tools (e.g., for metastasis prediction), the Prediction model Risk Of Bias Assessment Tool (PROBAST) was used. PROBAST assesses risk of bias across four domains: participants, predictors, outcomes, and analysis. Ratings were performed independently and followed guidance provided in the PROBAST handbook [15].

## Results

### Study selection and characteristics

#### Diagnostic studies

Thirteen eligible diagnostic studies published between 2012 and 2025 were identified. These studies evaluated AI-based models that aimed to discern uveal melanoma from ‘non-melanoma’, which could be either healthy eyes or benign lesions (i.e. nevi, congenital hypertrophy of the retinal pigment epithelium (CHRPE), other tumors, etc.) using imaging-based input, with or without other input like demographic and clinical data. Most studies were conducted in a single institution either in the United States or Europe, with sample sizes ranging from 123 to 4255 patients, and using 170 up to more than 27,000 images. Data was either collected from institutional datasets or gathered from public repositories. Most studies used retrospective data; only one study used a nationally pooled cohort [16]. Five studies explicitly addressed risk of patient repetition and performed patient-level data splits to avoid data leakage between training and test sets [16–20]. Still, earlier or lower-volume studies relied on small datasets with high likelihood of sample overlap or limited annotation protocols. Imaging modalities varied widely, with the most common being fundus photography in 10 studies, Iddir et al. used UWF and US [19], Tailor et al. utilized UWF, US, OCT, autofluorescence (AF) [20], and Jackson et al. used UWF and AF [18].

DL was employed in 10 of the 13 studies, with architectures including CNNs, ResNet variants, and general transformer-based models such as RETFound [18], a vision-language foundation model pre-trained on millions of retinal images and associated clinical text. These models are designed to learn transferable representations across diverse ophthalmic tasks by integrating visual and textual inputs during training. On the other hand, three studies used ML methods, including tree-based ensembles and lasso logistic regression [20, 21]. Only one study employed a semi-supervised learning approach (Tables 1 and 2) [22]. Information regarding patient characteristics, recruitment time period and institutions, and funding source can be seen on Supplementary Table 3.

**Table 1:** Baseline characteristics of diagnostic studies using machine learning or deep learning for the diagnosis of uveal melanoma

Authors (Year)	No. of patients	No. of images	Study design	Study objective	Reference standard
Tailor et al. (2025) [20]	2870	NR	Retrospective multicenter study	Predict risk of choroidal nevus transformation into melanoma using multimodal imaging	Growth ≥ 0.5 mm/24 mo or histopathology by expert
Ma et al. (2024) [23]	479 + 30 healthy controls	396 (training), 90 (testing), 30 (control)	Retrospective cohort	Automated lesion segmentation to differentiate uveal melanoma, choroidal nevi, and CHRPE from healthy eyes	Diagnosis by retina specialist
Dadzie et al. (2024) [17]	438	798	Retrospective cohort	Distinguish uveal melanoma from choroidal nevi using deep learning with colour fusion strategies	Diagnosis by retina specialist
Habeb et al. (2024) [24]	NR	400 (200 benign, 200 malignant)	Experimental study (simulation-based)	Classify ocular tumors (benign vs. malignant) using optimized deep learning models	Expert ophthalmologist-verified labels
Hoffmann et al. (2024) [25]	NR	762 (Clarus + Optos); 613 (Clarus only)	Retrospective cohort study	Distinguish highly malignant uveal melanoma from benign choroidal nevi using CFPs	Multimodal imaging (OCT, US, AF, expert review)
Santos et al. (2022) [26]	183 (150 healthy, 33 with UM)	2048 (after augmentation)	Experimental diagnostic system	Implement secure CAD system for uveal melanoma using CNN and image encryption	Diagnosis based on dataset from NY Eye Cancer Center
Santos et al. (2020) [27]	NR (healthy/unhealthy classified)	1622 (1424 healthy, 198 μm)	Experimental system	Detect uveal melanoma via iris image segmentation using fuzzy logic and neural networks	Diagnosis based on image features (e.g., Hu moments)
Iddir et al. (2023) [19]	221	223 (for both UWF and US)	Retrospective cohort	Predict thickness of uveal melanoma and distinguish from nevi using US and UWF images	Ground truth thickness by B-scan US and expert label
Sabazade et al. (2025) [16]	688	802	Retrospective multicenter cohort	Differentiate small choroidal melanomas from nevi using fundus photographs	Diagnosis by ocular oncologists with ≥ 5 year follow-up or multimodal imaging
Zabor et al. (2022) [21]	123 training; 240 external	NR	Retrospective cohort	Predict probability of small choroidal melanoma vs. nevus using clinical data	Documented growth or pathology (melanoma); ≥24 mo stability (nevus)
Jackson et al. (2025) [18]	4255	18,510 μm; 8,671 nevi; 1,192 healthy	Case-control study	Differentiate choroidal melanomas from nevi using a self-supervised model	Diagnosis by ocular oncologists (multimodal imaging + clinical exam)
Ganguly et al. (2019) [28]	NR	170	Experimental (proof-of-concept)	Develop a CNN-based method to detect eye melanoma from images	Labels by medical experts from NY Eye Cancer Center
Abdeljaber et al. (2021) [22]	NR	664 (original); 3600 (augmented)	Retrospective experimental study	Automate identification of six choroidal tumor types using semi-supervised learning on fundus images	Clinical diagnosis (UCSF)

**Table 2:** Results of diagnostic studies using machine learning or deep learning for the diagnosis of uveal melanoma

Authors (Year)	Imaging modality	Training validation/testing ratio	AI algorithms	Diagnostic accuracy
Tailor et al. (2025) [20]	Fundus photography, AF, OCT, B-scan US	80% training / 20% testing (Wills); external validation (Mayo)	XGBoost (SAINTS), LGBM, RF, Extra Tree	SAINTS AUC: 0.864 (Wills), 0.931 (Mayo); AUPRC: 0.244 (Wills), 0.533 (Mayo); Sensitivity: 0.635–0.869; Specificity: 0.981–0.984
Ma et al. (2024) [23]	Ultra-wide-field fundus photography (Optos)	396 training / 90 testing / 30 controls	DeepLabv3	Dice: 0.86 (UM), 0.81 (nevi), 0.85 (CHRPE); Sensitivity: 0.77 (UM), 0.67 (nevi), 0.77 (CHRPE); Specificity: 0.93 (healthy)
Dadzie et al. (2024) [17]	Ultra-widefield fundus photography (Optos)	5-fold cross-validation	DenseNet121 with transfer learning	AUC (step 2 binary): 0.9781 (UM vs. Nevi); Accuracy: 92.24%; F1: 0.8788; AUC (multiclass): 0.9467; Accuracy: 89.31%; Intermediate fusion best performer
Habeb et al. (2024) [24]	Fundus photography	10-fold cross-validation	VGG16, AlexNet, GoogLeNet with CSA-CFGD optimizer	Accuracy: 88.25% (VGG16), 90.75% (AlexNet), 91.75% (GoogLeNet); CSA-CFGD consistently outperformed baseline optimizers
Hoffmann et al. (2024) [25]	Color fundus photography	90% training / 10% validation; 74 images for binary test set	ResNet50-based CNN (HyperTumorEyeNet)	Binary (Clarus only): Accuracy 95.8%, AUC 1.00; Sensitivity 0.95; Specificity 0.95. Binary (Clarus+Optos): Accuracy 90.9%, AUC 0.99
Santos et al. (2022) [26]	RGB iris images	Train/Val/Test split not detailed; uses ResNet18 pretrained	ResNet18 (transfer learning)	Accuracy: 99% for UM detection (ResNet18); Encryption metrics: NPCR ~ 0.996, UACI ~ 0.296, Entropy ~ 7.954
Santos et al. (2020) [27]	RGB iris photographs	Train/test split not explicitly stated	Mamdani Fuzzy Inference System, Feedforward Neural Network	Fuzzy: Accuracy 76%, Sensitivity 88.8%, Precision 83.9%; Neural Net: Accuracy 96.04% with Feedforward Net
Iddir et al. (2023) [19]	B-scan US (US), Ultra-widefield fundus (UWF)	70% training / 30% testing	ResNet-18	UWF model: Sensitivity 77.3%, Specificity 96.9%, AUC 0.946; US model: Sensitivity 81.5%, Specificity 100%, AUC 0.941
Sabazade et al. (2025) [16]	Wide field and standard field fundus photography	495 train / 168 val / 86 test / 53 external test	Two-stage U-Net + shallow Random Forest	AUC: 0.88 (test and external); Sensitivity: 100% (test), 80% (external); Specificity: 74% (test), 81% (external); F-score: 0.72 (test), 0.71 (external)
Zabor et al. (2022) [21]	Imaging-derived variables (not images directly)	Internal bootstrap; external validation	Lasso logistic regression	AUC: 0.880 (training), 0.849 (bootstrap-corrected), 0.861 (external); key predictors: SRF, tumor height, orange pigment, optic disc distance
Jackson et al. (2025) [18]	Color and autofluorescence UWF photos	70% train / 10% val / 20% test (randomized, patient-level split)	RETFound (self-supervised DL)	Binary: AUROC 0.90, Accuracy 0.83, Sensitivity 0.79, Specificity 0.87; Tertiary: AUROC 0.92, Accuracy 0.82, F1-score 0.72
Ganguly et al. (2019) [28]	Fundus photographs	60% training / 40% testing	CNN	Accuracy: 91.76%, Sensitivity: 90%, Specificity: 95%
Abdeljaber et al. (2021) [22]	Fundus photographs (Optos, Topcon)	80% training / 20% testing	Multicon (SSL)	Accuracy: up to 97.2% (balanced dataset); ROC-AUC up to 1.00; Sensitivity and specificity up to 100% for minority classes in augmented dataset

#### Diagnostic performance

Diagnostic performance varied across studies, with reported sensitivities ranging from 62.96% to up to 90.00% and specificities from 80.56% to 100.00% [16, 19, 20]. Most models were designed for binary classification tasks, to differentiate uveal melanoma from benign lesions (often nevi), and consistently achieved higher diagnostic accuracy than models trained for multiclass classification.

In the meta-analysis, pooled sensitivity across all models was 78.21% [95% CI: 68.50-85.57] with considerable heterogeneity (Fig. 1A). Sensitivity estimates clustered around 78.86% [95% CI: 63.13–89.04] for fundus-based models, although they ranged widely. The greatest reported sensitivity was 90.00% by Ganguly et al. while the smallest one was that from Iddir (62.96%) [19, 28]. Studies using US inputs also achieved high sensitivity (up to 86.84%, and pooling up to 77.50%) for detection of key imaging features [20]. There was no significant statistical difference when comparing sensitivities obtained with fundus or US models (Fig. 1A).

Specificity estimates, pooled at 94.28% [95% CI: 85.29–97.91], were more consistent across models (Fig. 1B). The highest specificities were reported in US-based models and semi-supervised approaches. For instance, Iddir et al. reported perfect specificity (100.00%) for their US-based classification task while achieving only a 83.48% specificity in their fundus photography branch [19]. This discrepancy was consistent in the forest plot, with a pooled specificity of 98.16% [95% CI: 97.60–98.60] for US-based datasets and 87.70% [95% CI: 78.67–93.24] for photography-based datasets, with statistical significance (Fig. 1B).

**Fig. 2:**
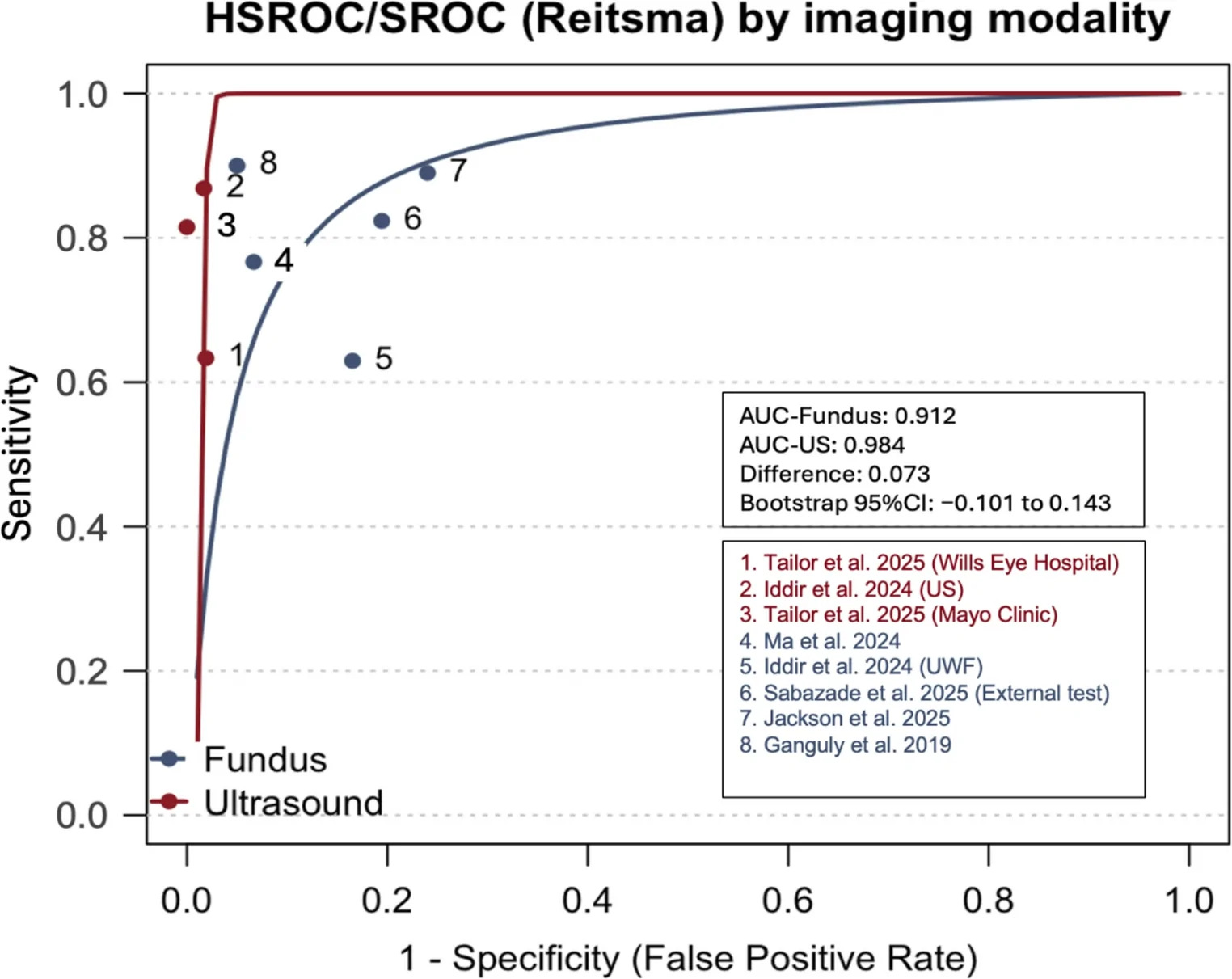
Hierarchical Summary Receiver Operating Characteristic (HSROC) curves stratified by imaging modality for machine learning–based uveal melanoma detection

HSROC analyses showed high overall discrimination by modality. AUC was 0.912 for fundus-based models and 0.984 for US-based models; although numerically higher with US, the AUC difference was not statistically clear (Δ0.073; bootstrap 95% CI − 0.101 to 0.143) (Fig. 2).

**Fig. 3:**
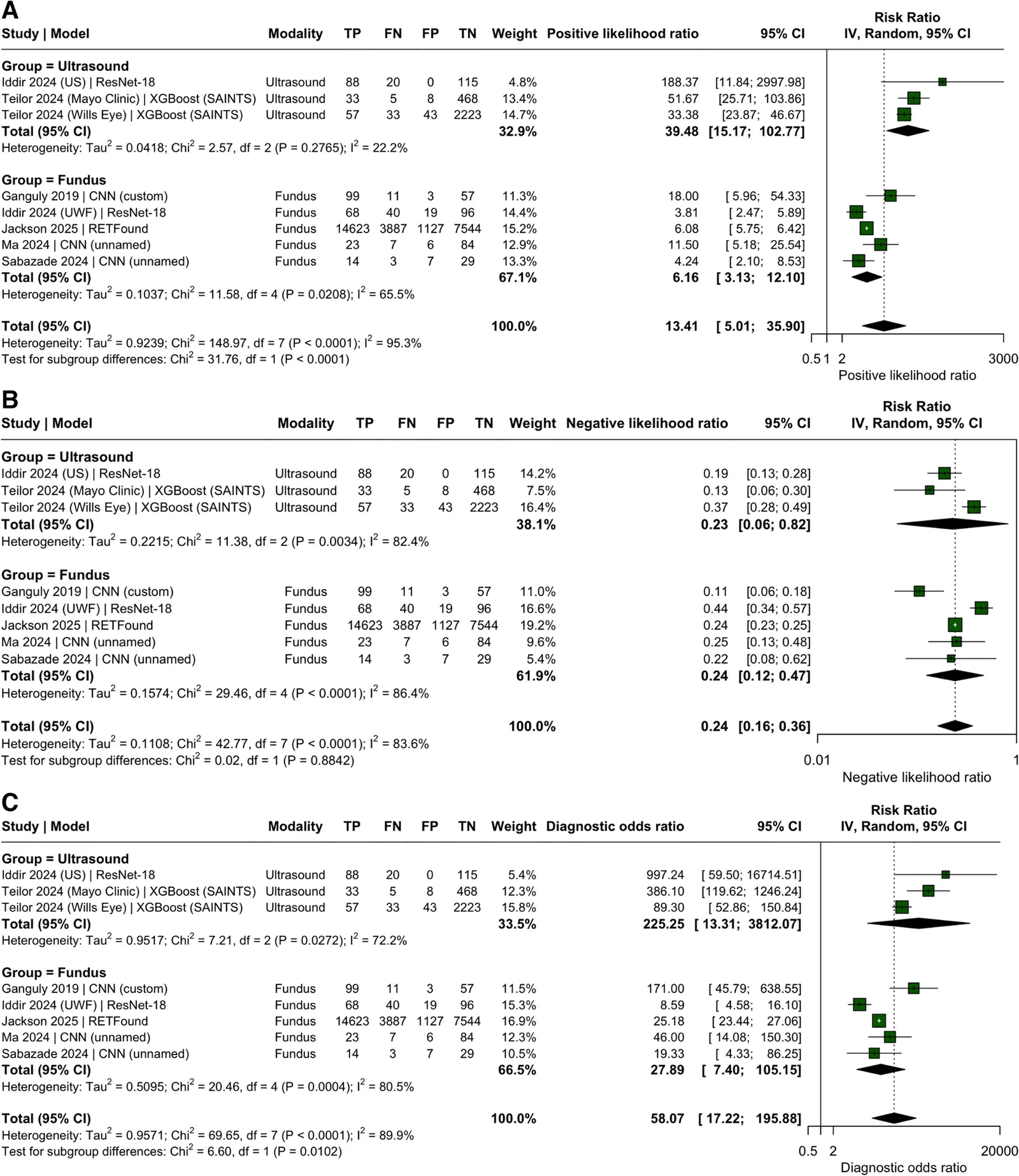
Subgroup analysis by imaging modality used as input in the training or validation model for (**A**) Positive likelihood ratio (**B**) Negative likelihood ratio and (**C**) Diagnostics odds ratio

By contrast, modality differences were most pronounced for LR+, with substantially higher values in US-based models (39.48 [15.17–102.77]) than fundus-based models (6.16 [3.13–12.10]; *p* < 0.0001) (Fig. 3A). DOR, integrating all four cells of the 2 × 2 table, was also higher for US (225.25 [13.31–3812.07]) versus fundus models (27.89 [7.40–105.15]; *p* = 0.0102), while LR − was similar between modalities (0.23 vs. 0.24; *p* = 0.884) (Fig. 3B-C).

#### Validation approaches

All included studies reported internal validation, most commonly using hold-out test sets or k-fold cross-validation. In addition, three studies conducted external validation using independent datasets. One of these studies evaluated image-based DL models in an entirely independent patient cohort [20], while the other two tested clinical or semi-supervised models using unseen institutional datasets [16, 21].

Generally, the performance in external validation was comparable to, or even higher than, internal validation outcomes. For instance, Tailor et al. reported a pooled AUROC of 0.864 in internal testing, which increased to 0.931 in external validation using the Mayo Clinic cohort, possibly reflecting differences in transformation rates between cohorts [20]. Sabazade et al. showed only a marginal drop, from an internal AUC of 0.885 (after random forest enhancement) to 0.880 in external testing, with balanced sensitivity (80%) and specificity (81%) on a small nationwide cohort [16]. Zabor et al. demonstrated a consistent performance between internal (AUC 0.849 using bootstrap correction) and external validation (AUC 0.861 on a 240-patient tertiary dataset), though calibration was suboptimal in the external set, likely due to population differences [21].

#### Comparison against human experts and decision-making tools

The study by Sabazade et al. was the only one to do this head-to-head comparison with human observers. They developed a DL model trained to differentiate small choroidal melanomas from nevi using only fundus photographs, and systematically compared its performance against multiple groups of human observers as well as two established clinical scoring systems, MOLES and TFSOM-UHHD [16]. In the primary test cohort of 86 images, using an optimal decision threshold of 0.63, the algorithm achieved a sensitivity of 100% and specificity of 74%. When benchmarked against human observers, resident ophthalmologists reached 85% sensitivity and 63% specificity, while consultant ophthalmologists achieved 83% sensitivity and 78% specificity. Both groups of general ophthalmologists demonstrated significantly lower sensitivity than the algorithm (*P* = 0.04 and *P* = 0.006, respectively), but no significant differences in specificity (*P* = 0.08 and *P* = 0.36). By contrast, subspecialized ocular oncologists performed comparably, with a sensitivity of 98% and specificity of 70%, differences that were not statistically significant (*P* > 0.99 for both). When data from all ophthalmologists were aggregated, the algorithm still demonstrated superior sensitivity (*P* = 0.006) without a significant difference in specificity (*P* = 0.54). In an external test cohort of 53 images drawn from outside institutions, the algorithm reproduced its overall discriminatory ability, achieving the same AUC as in the primary cohort and delivering sensitivity and specificity of 80% and 81%, respectively. The algorithm was also directly evaluated against the MOLES and TFSOM-UHHD risk stratification systems in the test cohort. While these frameworks incorporate multimodal imaging such as OCT and ultrasonography to capture features like hollowness or subretinal fluid, the DL model relied exclusively on color fundus photographs. Despite this more limited input, the algorithm demonstrated a markedly higher discriminatory performance with an AUC of 0.88 (95% CI, 0.82–0.95) and an F-score of 0.72, compared to 0.77 (95% CI, 0.66–0.88) and an F-score of 0.56 for MOLES, and 0.67 (95% CI, 0.54–0.81) for TFSOM-UHHD (both *P* < 0.001 vs. AI). Sensitivity and specificity for these tools were not reported [16].

Hoffmann et al. reported similar results, with their model reaching an AUC of 1.0. However, it misclassified 4 of 33 naïve uveal melanomas (12.1%) as benign cases that MOLES correctly identified as probable melanomas. These discrepancies mainly involved borderline lesions, and the authors emphasized that MOLES benefits from multimodal input, while their model used only fundus images. This points to a potential pitfall of AI models used in a screening scenario where the MOLES tool is the gold standard due to its sensitivity [25].

None of the included studies reported inter-rater reliability metrics or formal superiority testing between AI and clinicians.

#### AI architectures and model types

Across the 13 included studies, DL approaches predominated, with 10 studies implementing CNNs or related architectures. These included standard CNNs, advanced architectures such as ResNet, DenseNet, GoogLeNet, U-Net; and transformer-based models. Only Tailor et al., Zabor et al., and Santos et al. used traditional ML models, such as XGBoost, logistic regression, and fuzzy logic neural network, respectively [20, 21, 26].

Model choice often followed the data, but more importantly, it followed the question. When the aim was simply to classify images (melanoma vs. not), researchers used CNNs trained on raw fundus or UWF inputs, no need for pixel-level labels. But when the goal was to extract quantitative details about the lesion, segmentation came into play. Sabazade et al. first segmented tumors from images, then computed features like area, brightness, and shape. These were fed into a random forest, with no clinical variables, just image-derived metrics [16]. Meanwhile, Abdeljaber et al. tackled the problem of limited annotations by using semi-supervised learning: combining a small set of labeled images with many unlabeled ones. Through pseudo-labeling and augmentation, the model iteratively improved its own training data, achieving strong performance with minimal expert input [22].

### Prognostic studies

Six studies evaluated ML models for prognostic tasks in UM cases, including prediction of metastasis, chance of survival, enucleation, or final visual acuity. Sample sizes ranged from 424 to up to 4,600 patients, with follow-up periods from a minimum of 2 years to over 15 years (Table 3). Only one study performed direct analysis of the B-scan images [29] while the rest extracted structured variables such as tumor thickness.

**Table 3:** Baseline characteristics of prognostic studies using machine learning or deep learning for the diagnosis of uveal melanoma

Authors (Year)	No. of patients	Study design	Study objective	Training validation	AI algorithms	Prognostic accuracy
Vaquero-Garcia et al. (2017) [30]	1227	Retrospective prognostic model study	Predict risk of metastasis within 48 months using clinical and genomic features	10 × 10-fold cross-validation (797 labeled)	Logistic regression	AUC: 80% (chromosomes), 83% (clinical), 85% (combined)
Serghiou et al. (2019) [31]	1022	Prospective cohort	Predict visual acuity and ocular conservation after PBRT for choroidal melanoma	Cross-validation only	Elastic Net, Gradient Boosting, Deep NN, Bayesian	VA: R² = 28% (GBM); Enucleation: AUC = 80% (Elastic Net); top features: tumor thickness, radiation to macula, TNM stage
Luo et al. (2022) [29]	454 (death model), 424 (metastasis model)	Retrospective cohort	Predict 4-year risk of death and metastasis after plaque brachytherapy using US follow-up data	4-fold stratified cross-validation	Random Forest	AUC (death): 0.708 (1 FU), 0.883 (3 FU); AUC (metastasis): 0.730 (1 FU), 0.846 (3 FU); Accuracy: 83.02% (death), 79.48% (metastasis); Sensitivity: up to 80%
Wu et al. (2024) [32]	4189	Retrospective observational study	Predict distant metastasis in uveal melanoma using SEER data and ML	70% training / 30% internal test	Multilayer Perceptron (MLP)	AUC: 0.876; Precision: 0.788; Accuracy: 0.788; PRAUC: 0.788; Sensitivity: 0.784; Specificity: 0.862
Chen et al. (2022) [33]	1553	Retrospective prognostic study	Predict 2-year metastasis and 2-year death in Chinese UM patients using ML	4-fold cross-validation	Random Forest	UMDeath AUC: 0.87; UMMetastasis AUC: 0.84; High sensitivity and specificity (95% CI in Supp. Tables)
Firestone et al. (2023) [34]	4600	Retrospective registry-based study (SEER)	Predict 5-year and 10-year survival in choroidal melanoma	Not specified (train/test split used)	Multilayer Perceptron (MLP)	5-year AUC: 85.1%; Accuracy: 75.6% (test set); 10-year AUC: 92.1%; Accuracy: 87.1% (test set)

The majority of studies aimed to predict distant metastasis or all-cause mortality, with AUC values generally ranging from 0.71 to 0.92. The largest studies using SEER data achieved strong performance for 10-year survival and distant metastasis prediction respectively using multilayer perceptron models, with Firestone et al. achieving the highest AUC of these studies [32, 34]. Luo et al. demonstrated improved accuracy for both death and metastasis prediction when incorporating longitudinal US data at multiple follow-ups, reaching an AUC of 0.883 for mortality, higher than AUC within the same study with shorter follow-ups [29]. Serghiou et al. evaluated enucleation and visual outcomes after proton therapy, reporting better predictive performance for enucleation (AUC 0.80) than for final visual acuity (R² 28%) [31].

In single-center cohorts, models developed using Random Forest and logistic regression incorporated detailed imaging, clinical and chromosomal features to the model, achieving AUCs in the range of 80–87% [30, 33]. The PRiMeUM model developed by Vaquero-García et al. allowed for a personalized 48-month hepatic metastasis risk estimation and remains one of the few tools currently publicly available. The inclusion of dynamic post-treatment features, represents a notable advance in prognostication [30].

Across studies, common predictors included tumor thickness, diameter, AJCC stage, and patient age, while the inclusion of treatment sequence, imaging response, and sociodemographic factors varied widely. All studies were internally validated, and none conducted prospective or external validation.

Socioeconomic and demographic features emerged as relevant predictors in the SEER-based models, though these variables are often unavailable in prospective clinical workflows. Notably, models that performed well, particularly those predicting 10-year survival, frequently did so without access to molecular or histopathologic data, which limits biological granularity but may support broader applicability [32, 34].

### Quality assessment

The QUADAS-2 evaluation revealed common methodological issues, especially in the Index Test and Reference Standard domains. Eleven of thirteen studies had either “High” risk or “Some Concerns” in the Index Test domain, mainly due to missing pre-specified thresholds and lack of blinding during model evaluation. Studies like Habeb 2021, Santos 2020/2022, Ganguly, and Abdeljaber were rated High Risk for testing on the same dataset used for training and unclear evaluation procedures [22, 24, 26, 28]. Only Sabazade et al. and Zabor et al. clearly reported thresholds and blinded assessments [16, 21].

In the Reference Standard domain, several studies lacked clarity on how lesions were labeled, leading to High Risk assessments [22, 26, 28]. In contrast, other studies used validated standards like expert-confirmed growth or biopsy [16, 20, 21]. Patient Selection varied: studies with real-world cohorts and clear inclusion criteria were rated Low Risk, while small or poorly documented datasets led to High Risk ratings [16, 19–21, 23]. Flow and Timing was generally sound, though some older or less detailed studies received “Some Concerns.” (Supplementary Fig. 1).

All assessed prognostic studies with the PROBAST tool demonstrated low risk of bias in Participants, Predictors, and Outcome domains, reflecting robust cohort selection, clear variable definitions, and objective outcome measurement. However, all were rated as “Some Concerns” in the Analysis domain due to similar limitations. All studies lacked external validation, relying only on internal cross-validation or train/test splits, raising concerns about generalizability. Additionally, calibration metrics were often missing or incomplete, and none reported decision curve analysis to assess clinical usefulness.

## Discussion

This is the first study to systematically quantify the diagnostic performance of AI models for UM, providing pooled estimates of sensitivity and specificity and enabling formal subgroup comparisons by imaging modality. Across 13 diagnostic studies, pooled sensitivity and specificity were 78.21% [95% CI: 68.50–85.57] and 94.28% [95% CI: 85.29–97.91]. Stratified analyses showed similar sensitivity for US (77.50% [95% CI: 38.70–94.95]) and fundus models (78.86% [95% CI: 63.13–89.04]), but higher specificity for US (98.16% [95% CI: 97.60–98.60]) versus fundus (87.70% [95% CI: 78.67–93.24]). While HSROC AUC was high for both modalities (0.912 fundus vs. 0.984 US; Δ0.073, bootstrap 95% CI − 0.101 to 0.143), the clearest separation was observed in LR+ (39.48 vs. 6.16; *p* < 0.0001) and DOR (225.25 vs. 27.89; *p* = 0.0102), with comparable LR−. Heterogeneity remained moderate to high, particularly for fundus sensitivity (I² = 83%), reinforcing that US-derived depth and internal reflectivity cues may reduce false positives, whereas fundus-based approaches retain practical advantages for scalable screening workflows.

The results show that AI models have promising results when distinguishing uveal melanoma from choroidal nevi and other lesions. These findings are broadly consistent with those of Dadzie et al., who also noted that DL models excel when paired with well-annotated and diverse imaging datasets [35].

Most included studies used DL approaches, particularly convolutional neural networks, trained on fundus photographs or multimodal combinations including US and AF. Transformer-based architectures and semi-supervised learning models were less common but showed promising results in two studies [18, 22]. High-performing models often leveraged multimodal inputs and internal dataset curation, and externally validated studies, although fewer, still demonstrated robust performance, with AUCs ranging from 0.86 to 0.93.

Compared to traditional clinical scoring systems such as MOLES or TFSOM-UHHD, AI models demonstrated a more favorable balance between sensitivity and specificity. Several models outperformed or matched general ophthalmologists in retrospective evaluations. While the recent review by Dadzie et al. provided an important overview of developments in this space, our study extends this work by offering a quantitative analysis of the different modalities for UM detection [35].

### Foundational models

New large AI models such as RETFound [36] and VisionFM [37] could help address the main challenges in imaging-based machine learning for uveal melanoma: poor generalizability, inconsistent methods, and the lack of real-world testing. Unlike traditional deep learning models that are trained on small, single-center datasets, these foundation models are trained on millions of diverse eye images using self-supervised or multimodal learning. RETFound learns detailed retinal features that can be adapted to rare diseases like uveal melanoma, improving performance even when only a small number of labeled images are available. VisionFM goes further by learning from multiple imaging types, such as fundus, ultrasound, and OCT, making it highly relevant for the multimodal imaging used in uveal melanoma diagnosis. Both models also include tools for explaining their decisions and estimating uncertainty, which could make AI pipelines more standardized, transparent, and easier to validate in clinical settings.

A practical way to use these models for uveal melanoma would begin with standardizing image data across hospitals and devices. Then, pretrained encoders, for example, RETFound for fundus images and VisionFM for multimodal data, can be fine-tuned on uveal melanoma datasets using patient-level data splits to avoid overlap. For prognostic tasks, longitudinal imaging and clinical data (such as tumor thickness and treatment history) can be added through a multimodal framework. The workflow should include explainable AI tools (like Grad-CAM [38] or SHAP [39]) and uncertainty estimation to improve safety and trust. Finally, prospective multicenter validation across different hospitals and clinics can test how well the models perform in diverse settings while using privacy-preserving data-sharing methods, such as training the model locally at each site and sharing only the results or model updates instead of patient data. This approach allows research groups to collaborate without moving or exposing sensitive patient information, making it safer and more practical to test and improve the models for real-world clinical use.

### Limitations

A major limitation in the diagnostic literature is the scarcity of robust external validation. Although internal performance was frequently high, generalizability across independent settings remains largely untested. Only four studies incorporated external validation, restricting assessment of transportability across institutions, devices, and patient populations. Heavy reliance on internal test sets raises concerns about overfitting, inadvertent data leakage (e.g., near-duplicate images of the same eye leaking across splits), and optimistic bias, compounded by inconsistent dataset partitioning (patient-level rather than image-level splits were not universal). Several studies also reported performance at the image level, treating multiple images from the same patient/eye as independent observations; this clustering can inflate the effective sample size and narrow confidence intervals, so precision may be overstated. Considerable heterogeneity persisted even after stratification by imaging modality, particularly for sensitivity among fundus-based models. Accordingly, pooled estimates should be interpreted as average performance across heterogeneous designs and case-mix rather than a single deployable benchmark.

Prognostic studies must be analyzed carefully. They showed high internal accuracy, but poor reproducibility and minimal or often absent external validation are a problem all these studies share. Many relied on secondary sources of information rather than confirmed metastatic outcomes, risking misclassification. Diverse endpoints and index times prevented meta-analysis, allowing only qualitative synthesis. Most reduced survival data to binary outcomes, rarely addressed competing risks or calibration, and were limited by small event numbers and inconsistent data handling. Thus, although prognostic studies are promising and show interesting results, their methodological heterogeneity and lack of external validation limit their interpretability and make them inapplicable in clinical practice at this time, prompting further research in this field.

This review faced several methodological limitations. The small number of eligible studies, reduced power for subgroup analyses. Incomplete reporting limited uniform data extraction, with many studies providing only AUC values. Heterogeneity from varied imaging modalities and analytic methods persisted despite subgroup analyses. Bias of primary studies also poses a challenge for this review; risk-of-bias scores point out high risk in the index test domain and also highlight inconsistent external validation across different studies, both things that may overestimate the performance of the tests conducted. Nevertheless, the consistency of high diagnostic accuracy across multiple independent ML and DL architectures suggests that these findings are robust and clinically meaningful, providing a solid foundation for future refinement. Publication bias may also be present, studies reporting underperforming models may be less likely to be published. This could lead to an overestimation of pooled diagnostic performance and should be considered when interpreting the summary estimates.

### Future directions

Future research should emphasize standardized imaging protocols, robust external validation, and prospective comparisons with clinicians to assess real-world impact. Transparency in model development, through open data, code, and pretrained weights, is essential for reproducibility. Advances should include multimodal and ensemble designs, supported by self-supervised and few-shot learning, to overcome data scarcity. Foundation models such as RETFound and VisionFM could further enhance generalizability, explainability, and cross-center consistency. Encouragingly, there is now a clear path toward improving AI performance in UM, with growing evidence that these systems can complement clinical expertise and extend diagnostic capability. With continued methodological rigor and clinical translation, AI tools are poised to become essential components of future ocular oncology practice.

## Supplementary Information

Below is the link to the electronic supplementary material.

Supplementary Material 1.

## Data Availability

Data available on request from the authors The data that support the findings of this study are available from the corresponding author, AB-G, upon reasonable request.
