# Supplementary material for: Imaging-based machine learning for the diagnosis and prognosis of uveal melanoma: a systematic review and meta analysis
Source: Graefes Arch Clin Exp Ophthalmol. 2026 Apr 18;264(8):2329–42. doi: 10.1007/s00417-026-07230-3 (PMC13490244; doi:10.1007/s00417-026-07230-3)
Supplement: Supplementary file 1 [file 417_2026_7230_MOESM1_ESM.docx]

## *Supplementary Table 1. PICO frameworks for diagnostic vs. prognostic ML models in uveal melanoma*

| Element | Diagnostic ML models | Prognostic ML models |
| --- | --- | --- |
| Population (P) | Adults with suspected uveal melanoma or indeterminate choroidal lesions (e.g., nevus vs melanoma) at initial presentation | Adults with a confirmed diagnosis of uveal melanoma after baseline staging |
| Intervention / Index test (I) | Machine learning / deep learning models applied to imaging inputs (fundus, UWF, OCT, US, autofluorescence) | Machine learning / deep learning models using imaging with or without clinical, histopathologic, and/or genomic data |
| Comparator (C) | Reference standards such as expert clinical diagnosis, documented growth on follow-up, or histopathology | Established prognostic factors (e.g., AJCC staging, gene expression profiling, cytogenetics, clinical variables) |
| Outcomes (O) | Diagnostic accuracy metrics: sensitivity, specificity, AUC, predictive values; correct classification of benign vs malignant | Prognostic accuracy metrics: discrimination (AUC/C-index), calibration, survival outcomes (e.g., metastasis-free survival, overall survival, risk of enucleation) |

## *Supplementaty Table 2. Generic search strategy used in all of the databases*

| Entity | (("uveal melanoma" OR "choroidal melanoma" OR "ocular melanoma" OR "intraocular melanoma" OR "uveal pigmented lesion*" OR "choroidal pigmented lesion*" OR "intraocular pigmented lesion*" OR "ocular nevus" OR "choroidal nevus" OR "uveal nevus" OR "ocular nevi" OR "choroidal nevi" OR "uveal nevi" OR “ocular tumor” OR “eye tumor”)) |
| --- | --- |
| Boolean operator | AND |
| Intervention | (("machine learning" OR "artificial intelligence" OR "deep learning" OR "neural network*" OR "neural network*" OR "support vector machine*" OR "random forest" OR "supervised learning" OR "unsupervised learning" OR "computer vision")) |

## *Supplementaty Table 3. Institutions, recruitment years, funding and population baseline characteristics*

| Authors (years) | Population | Recrutiment institutions | Recruitment years | Funding |
| --- | --- | --- | --- | --- |
| Tailor et al. (2024) | 2,870 nevi; age 71.1±16.0 (Wills), 65.8±16.7 (Mayo); female ~63% / 62% | Ocular Oncology Service, Wills Eye Hospital (Philadelphia, PA); Mayo Clinic Rochester (Rochester, MN) | 2007–2017; 2015–2023 | NCI P30 CA015083; NCATS KL2 TR002379; Hoeft Career Development Award Fund |
| Ma et al. (2024) | 479 pts/529 lesions (+30 fellow eyes); age mean±SD: UM 68.7±13.2, nevi 65.2±16.0; sex (M/F) UM 73/78, nevi 81/156 | University of Illinois Chicago (UIC) Ear and Eye Infirmary | 2013–2024 | NEI P30 EY001792; RPB; VitreoRetinal Surgery Foundation; NCATS UL1TR002003 |
| Dadzie et al. (2024) | 438 pts (UM 157; nevus 281); age mean: 67.5 vs 63.2; White: 84% vs 64% | University of Illinois Chicago (UIC) eye clinic | 2010–2023 | NEI (R01 EY023522, R01 EY029673, R01 EY030101, R01 EY030842, P30EY001792); RPB; Richard & Loan Hill Endowment |
| Habeb et al. (2024) | 400 fundus images (200 benign; 200 malignant tumor patches) | Retina Image Bank (American Society of Retina Specialists); global image-sharing platform contributed by ophthalmologists/photographers worldwide | Not reported | National Natural Science Foundation of China (62172152) |
| Hoffmann et al. (2024) | 762 cases with choroidal melanocytic lesions (nevus; treatment-naïve melanoma; irradiated melanoma) | Ocular Oncology Department, Charité – Universitätsmedizin Berlin (Charité University Hospital Berlin), Germany | 2010–2023 | None reported (no external funding) |
| Santos-Bustos et al. (2022) | 183 images (150 healthy; 33 UM); augmented to 2,048 | Dr. Paul T. Finger, MD; New York Eye Cancer Center; Institute of Automation, Chinese Academy of Sciences | Not reported (accessed 2021) | Self-funded |
| Santos & Espitia (2020) | 1,622 images (UM 198; non-UM 1,424) | Facultad de Ingeniería, Universidad Distrital Francisco José de Caldas (Bogotá, Colombia); Dr. Paul T. Finger, MD; New York Eye Cancer Center | Not reported | Not specified (institutional support acknowledged) |
| Iddir et al. (2023) | 223 eyes/221 pts (nevus 115; UM 108) | Eye clinic, University of Illinois at Chicago (UIC) | 2010–2022 | Research to Prevent Blindness (UIC Dept. Ophthalmology & Visual Sciences); NCATS/NIH UL1TR002003 (editorial support) |
| Sabazade et al. (2024) | 688 pts; 802 images (nevus 73%, melanoma 27%); sex: 56% F / 44% M | Ocular Oncology Service, St. Erik Eye Hospital (Stockholm, Sweden; national referral center); external test images from other clinics across Sweden | ≥2010 | Swedish Society of Medicine (SLS-971390); Region Stockholm (FoUI-981345); Swedish Cancer Society (23 2613 Fk); Swedish Eye Foundation (2023-05-04) |
| Zabor et al. (2022) | Train: 123 pts (melanoma 61; nevus 62). External val: 240 pts (melanoma 11; nevus 229) | Training: Cole Eye Institute, Cleveland Clinic (tertiary ophthalmology clinic); Validation: separate tertiary ophthalmology clinic (name not reported) | Train 2010–2018; Val 1997–2001 | Research to Prevent Blindness (unrestricted grant to Cole Eye Institute) |
| Jackson et al. (2025) | 4,255 pts (melanoma 2,073; nevi 1,698; healthy 484); 28,373 images | Liverpool Ocular Oncology Center, Liverpool University Hospitals NHS Foundation Trust (UK) | 1995–2020 | North West Cancer Research; NIHR AI Health & Care Award (partial) |
| Ganguly et al. (2019) | 170 images (melanoma 110; normal 60) | New York Eye Cancer Center database; Meghnad Saha Institute of Technology, Electrical Engineering Dept. (Kolkata, India) | Not reported | Not reported |
| Abdeljaber et al. (2021) | 664 fundus images (6 tumor classes); augmented to 3,600 | Ocular Oncology Service, University of California San Francisco (UCSF) | Not reported | NSF (DMS-1737978, DGE-2039542, OAC-1828467, OAC-1931541, DGE-1906630); IBM faculty award |
| Vaquero-Garcia et al. (2017) | 1,227 UM cases (met-free 593; met+ 204; unlabeled 430) | Ocular Oncology Service, Wills Eye Hospital (Philadelphia, PA); Genetic Diagnostic Laboratory, University of Pennsylvania | 1985–2016 | NIA R01 AG046544; Penn IBI Pilot Grant; NCI 5R21CA181935 |
| Serghiou et al. (2019) | 1,022 adults with choroidal malignant melanoma; median age 59 | Ocular Oncology Service, University of California San Francisco (UCSF); Oxford Eye Hospital, University of Oxford (UK) | Not reported | NEI (EY002162; 1K23EY027466); Research to Prevent Blindness (unrestricted + CDA); That Man May See, Inc. |
| Luo et al. (2022) | 454 adults with UM; age mean±SD 46.3±11.7; male 210 (46.3%) | Beijing Tongren Eye Center; Beijing Key Lab of Intraocular Tumor Dx/Tx; Beijing Tongren Hospital, Capital Medical University (Beijing, China) | 2007–2016 | NSFC 82101180; Beijing NSF 7204245; plus multiple Beijing municipal/education/hospital grants |
| Wu et al. (2024) | 4,189 UM; male 51% (2,128); White 98% | SEER database (retrospective; 22 regions) | 2000–2020 | None reported |
| Firestone et al. (2023) | 4,600 US choroidal melanoma cases (SEER; ICD-O-3) | SEER database (nationwide US cancer registry); author affiliations: Dean McGee Eye Institute; Baylor College of Medicine; Univ. of Tennessee Health Science Center | 2000–2018 | None reported (abstract); note: author support from NEI & BrightFocus Foundation |

NCI = National Cancer Institute; NCATS = National Center for Advancing Translational Sciences; NEI = National Eye Institute; RPB = Research to Prevent Blindness; UM = uveal melanoma; UIC = University of Illinois Chicago; NIH = National Institutes of Health; SD = standard deviation; M = male; F = female; SLS = Swedish Society of Medicine; FoUI = Region Stockholm Research, Development and Innovation; NIHR = National Institute for Health and Care Research; AI = artificial intelligence; NSF = National Science Foundation; NIA = National Institute on Aging; IBI = Institute for Biomedical Informatics; SEER = Surveillance, Epidemiology, and End Results; ICD-O-3 = International Classification of Diseases for Oncology, Third Edition; Dx/Tx = diagnosis/treatment; NSFC = National Natural Science Foundation of China; CDA = Career Development Award; met-free = metastasis-free; met+ = metastasis-positive; val = validation.

## *Supplementary Figure 1. PRISMA Diagram of systematic search, screening and included studies*

## *Supplementary figure 2. QUADAS-2 quality assessment (A) table and (B) summary metrics*
